# 作者更正

**Published:** 2024-01

由于作者疏忽，刊登于《中华血液学杂志》2023年第11期第963–968页“新型BTK抑制剂治疗慢性淋巴细胞白血病临床研究进展”一文遗漏基金相关信息。更正如下，基金项目：江苏省科教能力提升工程（ZDXK202209）。特此更正。

本刊编辑部

